# Effect of *Lactobacillus plantarum* Biofilms on the Adhesion of *Escherichia coli* to Urinary Tract Devices

**DOI:** 10.3390/antibiotics10080966

**Published:** 2021-08-11

**Authors:** Fábio M. Carvalho, Rita Teixeira-Santos, Filipe J. M. Mergulhão, Luciana C. Gomes

**Affiliations:** LEPABE—Laboratory for Process Engineering, Environment, Biotechnology and Energy, Faculty of Engineering, University of Porto, Rua Dr. Roberto Frias, 4200-465 Porto, Portugal; up201502963@fe.up.pt (F.M.C.); ritadtsantos@fe.up.pt (R.T.-S.); filipem@fe.up.pt (F.J.M.M.)

**Keywords:** biofilm, probiotic, *Lactobacillus plantarum*, antibiofilm activity, urinary tract devices, *Escherichia coli*

## Abstract

Novel technologies to prevent biofilm formation on urinary tract devices (UTDs) are continually being developed, with the ultimate purpose of reducing the incidence of urinary infections. Probiotics have been described as having the ability to displace adhering uropathogens and inhibit microbial adhesion to UTD materials. This work aimed to evaluate the effect of pre-established *Lactobacillus plantarum* biofilms on the adhesion of *Escherichia coli* to medical-grade silicone. The optimal growth conditions of lactobacilli biofilms on silicone were first assessed in 12-well plates. Then, biofilms of *L. plantarum* were placed in contact with *E. coli* suspensions for up to 24 h under quasi-static conditions. Biofilm monitoring was performed by determining the number of culturable cells and by confocal laser scanning microscopy (CLSM). Results showed significant reductions of 76%, 77% and 99% in *E. coli* culturability after exposure to *L. plantarum* biofilms for 3, 6 and 12 h, respectively, corroborating the CLSM analysis. The interactions between microbial cell surfaces and the silicone surface with and without *L. plantarum* biofilms were also characterized using contact angle measurements, where *E. coli* was shown to be thermodynamically less prone to adhere to *L. plantarum* biofilms than to silicone. Thus, this study suggests the use of probiotic cells as potential antibiofilm agents for urinary tract applications.

## 1. Introduction

Biofilms, defined as communities of microorganisms attached to inert or living surfaces and surrounded by a self-synthetized matrix of extracellular polymeric substances, are a form that microbes adopt to expand metabolic cooperation and gain protection from hostile environmental conditions, such as immune responses and antimicrobial treatments [1,2]. This constitutes a challenge in treating biofilm-associated infections and is particularly severe since the presence of biofilms accounts for about 80% of human microbial infections [3,4]. Bacteria in biofilms are often hard to eradicate by antibiotics, the continuous administration of which leads to high rates of antibiotic resistance by pathogens [5].

Healthcare-associated urinary tract infections (UTIs) have a prevalence ranging from 13% in the USA and 20% in Europe, to up to 24% in developing countries [6,7], due to the common insertion of invasive urinary tract devices (UTDs), including urinary catheters and urethral stents [8,9]. UTIs have a great impact on patients’ safety and healthcare systems, causing high morbidity and mortality, increased hospitalization periods, and costly treatment [10,11,12]. UTDs are typically made of polymeric materials which, despite their attractive mechanical properties, are intrinsically prone to microbial adhesion and consequent biofilm development [13]. Additionally, the innate defense mechanisms of the urinary tract system might be weakened when a UTD is placed, making the microorganisms in the lumen of UTDs less susceptible to phagocytosis or the action of antimicrobial agents [4]. The majority of these infections are caused by *Escherichia coli*, *Klebsiella pneumonia*, *Pseudomonas aeruginosa*, *Staphylococcus aureus*, *Enterococcus faecalis*, and *Proteus mirabilis* [14,15,16]. However, *E. coli* has been described as the most common pathogen in short-term UTD biofilms, being able to invade and persist as biofilm-like aggregates inside bladder superficial cells [17].

In recent years, many advances have been made in the development of effective antimicrobial therapeutics to reduce the incidence of UTD infections. Novel strategies to prevent biofilm formation on UTDs include antimicrobial agents’ release, contact-killing, the inhibition of microbial adhesion, and disruption of the biofilm architecture [18,19]. However, although these approaches were shown to prevent adhesion and biofilm formation in the medical field, questions relating to bacterial resistance, cytotoxicity, biocompatibility, and long-term efficacy during catheterization require more extensive validation in future studies [11,19].

Probiotics were recently introduced as an interesting option to inhibit or delay the onset of biofilm formation in medical devices [20]. The activity of probiotics and/or their isolated metabolites against medical device-associated infections, including UTDs, was recently reviewed [21,22,23]. Probiotics are beneficial live microorganisms that, when ingested in sufficient numbers, produce health benefits for the host [24]. They are commonly known as non-pathogenic friendly bacteria and have received substantial attention because of their health-promoting properties, particularly in the digestive tract and immune system, possessing GRAS (generally regarded as safe) status [25]. The most commonly used probiotics are various species of lactic acid bacteria (LAB), which include *Lactobacillus*, *Bifidobacterium*, *Streptococcus* and *Lactococcus* [26,27]. *Lactobacillus* is one of the most important groups of the LAB genus [28]. It comprises a large heterogeneous group of Gram-positive, non-sporulating and facultative anaerobic or microaerophilic rod-shaped bacteria, including *Lactobacillus acidophilus*, *Lactobacillus casei*, *Lactobacillus fermentum*, *Lactobacillus plantarum,* and *Lactobacillus rhamnosus* [29,30]. They are nutritionally fastidious, requiring rich media to grow [31]. Metabolically, LAB are known to produce lactic acid as a major end-product of carbohydrate fermentation and other metabolites [32,33,34]. The selection of probiotic organisms requires a systematic approach to infer their functional and beneficial properties. It is necessary to consider their ability to adhere to surfaces, produce inhibitory substances, and survive and grow under adverse conditions [31,33,35]. The effectiveness of probiotics is strain-specific, and each strain has multiple and diverse impacts on the host through different mechanisms [36]. These mechanisms include the production of antagonistic substances that generate a physiologically restrictive environment for pathogens, competition for surface area and nutrients, biofilm formation, and the prevention of quorum sensing (QS) [37,38,39].

Despite promising results in displacing adhering uropathogens and inhibiting bacterial adhesion to catheter materials [40,41,42,43,44,45,46,47,48], the application of probiotics as a coating for UTDs remains barely explored. Since bacterial adhesion and biofilm growth are affected by the hydrodynamic forces around surfaces [49], and these forces vary between the upper and lower urinary tract, as well as on the inner and outer part of the device, it is important to consider this variability when setting an experimental assay of biofilm formation [9]. The experiments should also use biologically relevant fluids like pooled human urine or artificial urine [9]. Most of the antibiofilm assays are performed under static conditions [40,43,44,45,46], characteristic of the extraluminal side of UTDs [9], while just a small number of studies used the controlled hydrodynamic conditions [47,48] from intraluminal surfaces in the urethra, bladder or ureter [9]. On the other hand, only a couple of studies used nutritional conditions similar to those found in human urine [47,48].

Hence, the present study aimed to optimize the biofilm-forming capacity of a probiotic strain, *Lactobacillus plantarum*, and evaluate the ability of *L. plantarum* biofilms to prevent *E. coli* biofilm formation on silicone rubber. Additionally, the thermodynamic propensity of *E. coli* to adhere to silicone rubber with and without *L. plantarum* biofilms was determined. To the best of our knowledge, this is the first study using pre-formed probiotic biofilms to prevent the biofilm formation of pathogens, combining the effect of temperature, device surface, and nutritional and hydrodynamics conditions that mimic a specific urinary tract region. In this way, we have used more realistic conditions in order to increase the predictive value of this work.

## 2. Results

### 2.1. Biofilm-Forming Capacity of L. plantarum

**Figure 1:**
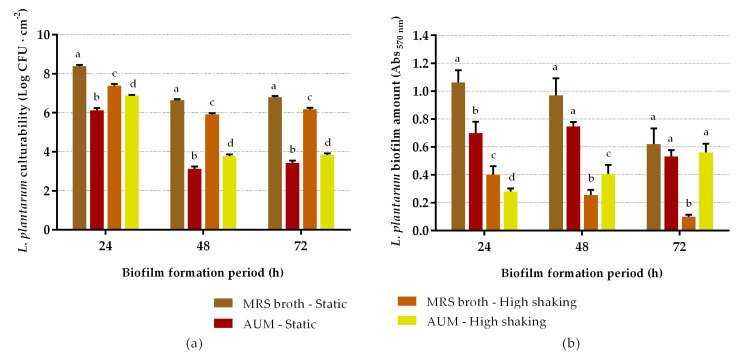
Culturability (**a**) and total biomass (**b**) of *L. plantarum* biofilms after 24, 48 and 72 h of development. Biofilms were formed on silicone in two different media (AUM and MRS broth) and at two hydrodynamic conditions (static and high shaking conditions). The data shown represent the mean and standard deviation (SD) of at least three independent experiments. Different lower-case letters indicate statistically significant differences between the same biofilm formation times (*p* < 0.05).

The ability of *L. plantarum* to adhere and form biofilms on silicone coupons was first evaluated by combining different hydrodynamic, nutritional, and temporal conditions. *L. plantarum* demonstrated a remarkable ability to adhere and establish biofilms on silicone (Figure 1). Looking at the effect of incubation time, cell culturability (Figure 1a) and biomass amount (Figure 1b) decreased over time, regardless of the growth medium and hydrodynamics. Biofilms that formed under static conditions presented significantly higher biomass amounts than those grown under high shaking conditions (*p* < 0.05; Figure 1b). Furthermore, *L. plantarum* biofilms formed in De Man, Rogosa and Sharpe (MRS) broth exhibited higher culturability and biomass than those formed in artificial urine medium (AUM).

**Figure 2:**
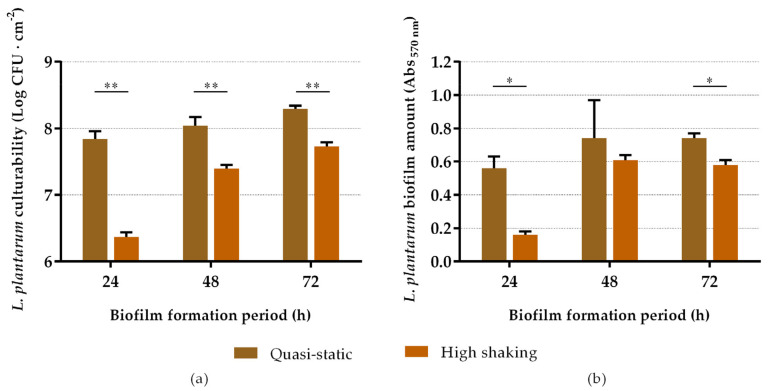
Culturability (**a**) and total biomass (**b**) of *L. plantarum* biofilms after 24, 48 and 72 h of development under quasi-static and high shaking conditions. Biofilms were formed on silicone coupons in MRS broth with medium replacement every day. The data present the mean and standard deviation (SD) of at least three independent experiments. Statistically significant differences between quasi-static and high shaking conditions were considered for *p*-values < 0.05 (* and ** indicate *p* < 0.05 and *p* < 0.01, respectively).

Then, a second screening was performed using MRS broth under different hydrodynamic and temporal conditions, replacing the culture medium every day to ascertain any possible nutritional limitations. As regards biofilm cell culturability (Figure 2a), it can be observed that it significantly increased over time (*p* < 0.05), regardless of the hydrodynamic condition, reaching the highest culturability levels after 72 h of incubation under quasi-static conditions, with 8.29 ± 0.05 log CFU·cm^−2^. Comparing the effect of agitation on biofilm formation, biofilms that formed under quasi-static conditions presented higher culturability values than those grown under more intense orbital shaking conditions (*p* < 0.01). The values ranged from 7.84 ± 0.12 to 8.29 ± 0.05 log CFU·cm^−2^ (with an average value of 8.06 log CFU·cm^−2^) for quasi-static conditions, and between 6.37 ± 0.07 and 7.73 ± 0.06 log CFU·cm^−2^ (with an average value of 7.17 log CFU·cm^−2^) for high shaking conditions. The results of biomass analysis were generally in agreement with those of culturability (Figure 2b). The Abs _570 nm_ values ranged from 0.56 ± 0.07 to 0.74 ± 0.03 for quasi-static conditions, and between 0.16 ± 0.02 and 0.61 ± 0.03 for high shaking conditions. There was an increase in biofilm biomass over time for both hydrodynamic conditions; however, for low agitation, the differences were not statistically significant (*p* > 0.05). These results allowed us to draw conclusions about the ideal conditions needed for the growth of robust *L. plantarum* biofilms to cover the silicone surfaces in the antibiofilm assays against *E. coli*. Although the culturability of 72 h biofilms was slightly higher than that of 48 h biofilms (8.29 ± 0.05 versus 8.04 ± 0.13 log CFU·cm^−2^; *p* = 0.012), the biomass amount was very similar (Abs _570 nm_ 0.74 ± 0.03 and 0.74 ± 0.23, respectively; *p* = 1.000). Thereby, in further assays, *L. plantarum* biofilms were formed for 48 h in MRS broth under quasi-static conditions with medium replacement.

### 2.2. Antibiofilm Assays

**Figure 3:**
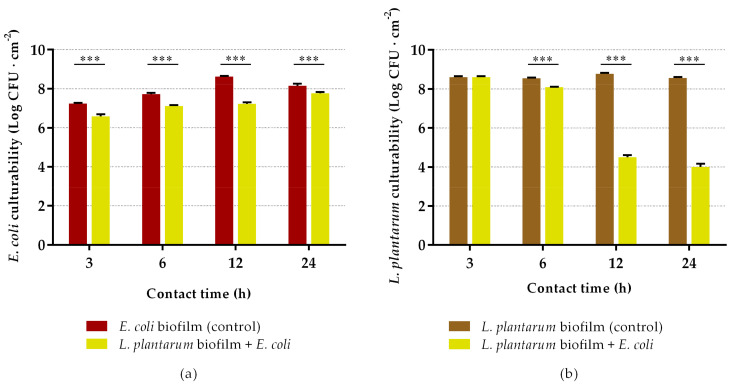
Culturability of *E. coli* (**a**) and *L. plantarum* (**b**) after 3, 6, 12 and 24 h of contact between the pre-formed probiotic biofilm and the pathogen. The biofilms of *L. plantarum* were formed for 48 h on silicone coupons under quasi-static conditions in MRS broth at 37 °C with medium replacement. *E. coli* was subsequently added to the wells for 3, 6, 12 and 24 h under quasi-static conditions in AUM at 37 °C. The culturability of *L. plantarum* and *E. coli* cells in the biofilm was determined by colony-forming units (CFU) counts. The data present the mean and standard deviation (SD) of at least three independent experiments. Statistically significant differences between the control and exposure were considered for *p*-values < 0.05 (*** indicates *p* < 0.001).

Having established the ideal conditions for biofilm formation, we proceeded to evaluate the ability of pre-established *L. plantarum* biofilms to inhibit the early development of *E. coli* biofilms. The results of culturable cells of *E. coli* and *L. plantarum* after 3, 6, 12 and 24 h of incubation are presented in Figure 3. As can be observed in Figure 3a, the number of *E. coli* culturable cells was significantly reduced over time when exposed to *L. plantarum* biofilms, in comparison to bare silicone (*p* < 0.001). Reductions of 76% and 77% were obtained after 3 and 6 h of incubation, respectively. The highest decrease in *E. coli* biofilm formation was achieved after 12 h of exposure, with a reduction of 99%, this being the most promising experimental time. However, when the contact between the bacterial strains was prolonged for 24 h, the ability of *L. plantarum* biofilms to inhibit the colonization of *E. coli* started to decrease (percentage of reduction: 60%). The culturability of *L. plantarum* in biofilms was also evaluated (Figure 3b) and the results showed a significant decrease over time, being more pronounced at 12 and 24 h with a 99.9% reduction when compared to control (*p* < 0.001). The presence of *L. plantarum* on the dual-species biofilms remained dominant during the first 6 h, where *L. plantarum* represented approximately 95% of the total biofilm population. However, after 12 h of contact, there was a marked decrease in the quantity of *L. plantarum* culturable cells in the biofilm.

**Figure 4:**
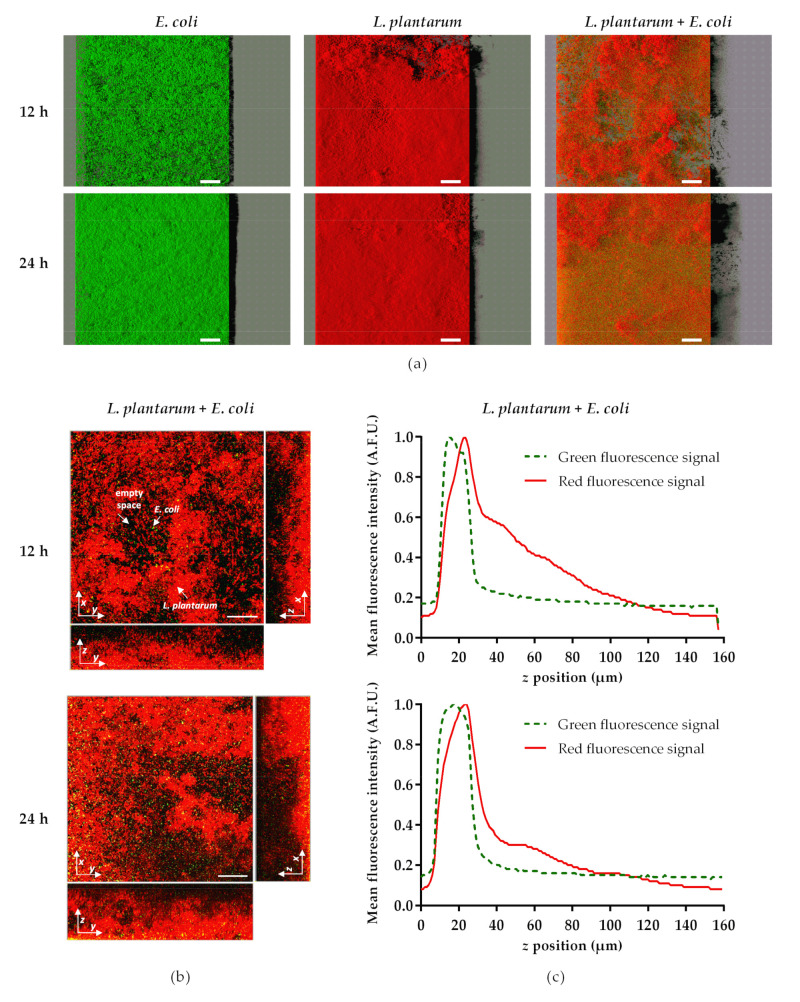
Spatial heterogeneity of 12- and 24-h biofilms from single- (*E. coli* CECT 434 GFP and *L. plantarum*) and dual-species (*L. plantarum* + *E. coli* CECT 434 GFP) cultures formed on silicone coupons: (**a**) aerial, three-dimensional (3D) view of the biofilms (shadow projection on the right); (**b**) section views of the CLSM images presented in (**a**) for the *L. plantarum* + *E. coli* CECT 434 GFP biofilms; and (**c**) distribution of normalized red and green fluorescence intensity values (arbitrary fluorescence units (A.F.U.)) along the vertical (*z*) biofilm position. The images in (**a**,**b**) were obtained from confocal *z*-stacks using IMARIS software. The white arrows in (**b**) indicate the presence of *E. coli* CECT 434 expressing GFP (labeled in green), *L. plantarum* (counterstained in red with Syto61), and empty spaces within the biofilm. Some yellow regions appear due to the red and green being superimposed. The white scale bars correspond to 50 µm in (**a**) and 100 µm in (**b**).

**Figure 5:**
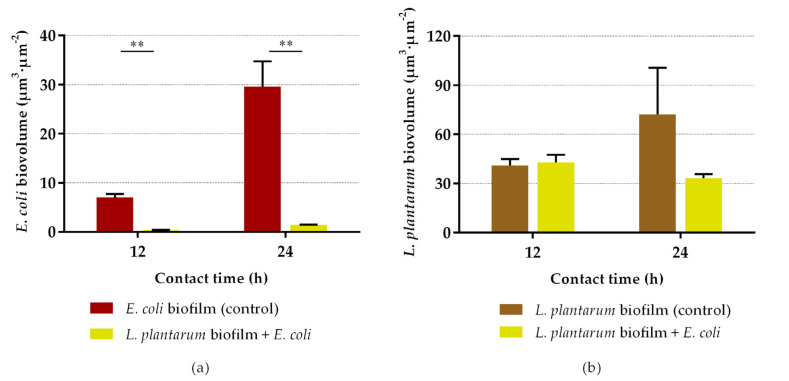
Biovolume of *E. coli* (**a**) and *L. plantarum* (**b**) after 12 and 24 h of contact between the pre-formed probiotic biofilm and the pathogen. This quantitative biofilm parameter was determined from confocal *z*-stacks using the plug-in COMSTAT2 associated with the ImageJ software. The data present the mean and standard deviation (SD) of at least five stacks of each sample. Statistically significant differences between the control and exposure were considered for *p*-values < 0.05 (** indicates *p* < 0.01).

In order to observe the spatial distribution and the heterogeneity of *E. coli* and *L. plantarum* populations in single- and dual-species biofilms, a confocal laser scanning microscopy (CLSM) analysis was performed after 12 and 24 h of exposure (Figure 4). The representative confocal images (Figure 4a) showed a marked reduction of *E. coli* colonization in dual-species biofilms, compared with *E. coli* single-species biofilms. In fact, while bare silicone surfaces were covered by dense *E. coli* biofilms (especially after 24 h of exposure, with a surface coverage of approximately 90%), only a few single cells of *E. coli,* heterogeneously distributed, were observed in the presence of *L. plantarum* biofilms. Looking at the vertical profiles of *L. plantarum* + *E. coli* biofilms (Figure 4b), these few bright cells of *E. coli* expressing green fluorescent protein (GFP) were mainly located in the interior regions of the biofilms, near the bottom, while *L. plantarum* cells (marked in red) were positioned all over the biofilm. This was confirmed by the quantitative results extracted from the *z*-stack acquisition (Figure 4c), which showed that *E. coli* cells that emitted the green signal occupied the bottom layer of the biofilm. This suggests that *E. coli* tends to penetrate the pre-established biofilms of *L. plantarum* and lodge at the bottom of the biofilm. Moreover, although mixed biofilms are mostly composed of *L. plantarum* (Figure 3, Figure 4 and Figure 5), the presence of empty spaces (black regions) in dual-species biofilms could be observed, in contrast to the dense single-species biofilms of *L. plantarum* covering the silicone surfaces (Figure 4a), corroborating the culturability results obtained for both bacteria.

**Figure 6:**
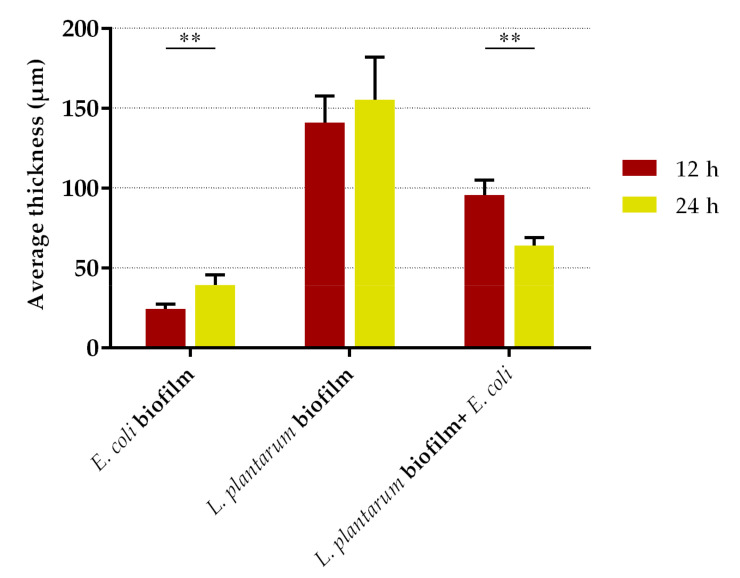
Thickness of single- (*E. coli* and *L. plantarum*) and dual-species (*L. plantarum* + *E. coli*) biofilms after 12 and 24 h of contact between the pre-formed probiotic biofilm and the pathogen. This quantitative biofilm parameter was determined from confocal *z*-stacks using the plug-in COMSTAT2 associated with the ImageJ software. The data present the mean and standard deviation (SD) of at least five stacks of each sample. Statistically significant differences between exposure times were considered for *p*-values < 0.05 (** indicates *p* < 0.01).

Regarding the quantitative CLSM analysis, *L. plantarum* biofilms significantly reduced the biovolume of *E. coli* by 95% at 12 and 24 h (*p* < 0.01; Figure 5a). Conversely to Figure 3b, where there was a decrease in the culturability of *L. plantarum* from single- to dual-species biofilm at 12 h, in Figure 5b the biovolume of *L. plantarum* at 12 h showed no significant differences between the control and exposure groups (*p* = 0.564). Likewise, the biovolume of *L. plantarum* in single-species biofilms at 24 h was higher than at 12 h (*p* = 0.064; Figure 5b), while no differences were found in culturability between the same samples (Figure 3b). The biofilm thickness was also assessed (Figure 6). The thickness of dual-species biofilms was reduced from 96 µm at 12 h to 64 µm at 24 h (*p* < 0.001). On the other hand, the thickness of single-species biofilms of *L. plantarum* was maintained at approximately constant (*p* = 0.584), and the thickness of single-species biofilms of *E. coli* increased from 24 µm to 39 µm between 12 and 24 h (*p* < 0.01).

### 2.3. Thermodynamic Surface Properties

The physicochemical interaction between *E. coli* CECT 434 GFP and silicone with and without *L. plantarum* biofilms were assessed through contact angle measurements and the calculation of the respective free energy ($\Delta G_{sws}$) (Table 1). The water contact angle ($\theta_{w})$ and the $\Delta G_{sws}$ can be used as indicators of the hydrophobicity of a surface. The$\;\theta_{w}$ of 107.1 ± 4.9° and $\Delta G_{sws}$ of −62.4 mJ·m^−2^ of silicone indicated its hydrophobic character. Conversely, *L. plantarum* biofilms revealed a hydrophilic character, with a $\theta_{w}$ of 34.9 ± 2.8° and $\Delta G_{sws}$ of 34.3 mJ·m^−2^. *E. coli* also demonstrated a hydrophilic character ($\theta_{w}$ = 19.4 ± 2.2° and $\Delta G_{sws}$ = 131.2 mJ·m^−2^), as is similar to previous studies with the same bacterial species [50].

Bacterial adhesion is favorable if the interactions lead to negative values of the free energy of adhesion$\;(\Delta G_{adhesion}$) [51]. In this study, since the $\Delta G_{adhesion}$ between *E. coli* and the two surfaces (bare silicone and silicone coated with *L. plantarum* biofilm) had a positive value (Table 1), *E. coli* adhesion to silicone and *L. plantarum* biofilms was thermodynamically unfavorable. Furthermore, the $\Delta G_{adhesion}$ between *E. coli* and *L. plantarum* biofilms was higher than that between *E. coli* and bare silicone (84.0 versus 36.1 mJ·m^−2^), which means that *E. coli* is less prone to adhere to *L. plantarum* biofilms than to silicone, from a purely thermodynamic perspective.

## 3. Discussion

With the widespread use of urinary tract devices, the formation of microbial biofilms on their surfaces contributes to the increasing number of urinary infections. Recently, some studies have proposed the use of probiotics and their metabolites as a reliable option to inhibit pathogenic biofilm growth and/or to disperse pre-formed biofilms in human subjects [23]. Although probiotics may exert different effects against the activity of pathogenic bacteria (displacement, exclusion, and competition), avoiding the initial attachment of pathogens by coating the surfaces seems to be the best strategy to fight biofilm-based infections [22]. Thus, in this work, the potential of *L. plantarum* to prevent biofilm formation on UTDs was evaluated, following an exclusion strategy. This strategy consists of the formation of *L. plantarum* biofilms, followed by exposure to *E. coli* suspensions, in order to evaluate the ability of *L. plantarum* as a protective barrier to fence off and retard pathogen colonization. This strategy is relevant since various exometabolites of *Lactobacillus* strains may inhibit biofilm formation by interfering with the initial attachment of pathogens [52,53]. Compared to other coating strategies, the use of non-pathogenic cells to coat medical devices may be advantageous because the coating is alive. This allows for the self-renewal of the anti-pathogenic activity, whereas conventional coatings eventually become covered by biomass, which may reduce their effectiveness and lead to the loss of beneficial properties over time [40]. This approach also confers a sustainable antimicrobial pathway to preventing UTIs without inducing bacterial resistance [19].

The first goal of this study was to optimize the cultivation conditions for the formation of robust *L. plantarum* biofilms on silicone surfaces. *L. plantarum* demonstrated a high biofilm-forming ability in MRS broth, as expected, since MRS broth has been described as an appropriate growth medium for *Lactobacillus* strains [54]. *L. plantarum* culturability and biomass amount decreased over time, regardless of the growth medium and hydrodynamic conditions. This tendency may be associated with nutritional limitations inside the wells. Since the culture medium was not replaced during these first sets of experiments, most of the nutrients may have been depleted, leading to limitations in probiotic growth. Moreover, biofilms formed under the static mode presented significantly higher biomass amounts than those grown under high shaking conditions. However, we believe that these static biofilms consisted of poorly adhered cells as a result of sedimentation [55]. This characteristic was observed while unsticking the silicone coupons from the wells, where the static-formed biofilm often disintegrated. In order to overcome this issue, a second screening of the biofilm-forming capacity of *L. plantarum* was performed; a quasi-static condition was tested in order to obtain both the higher biomass content characteristic from static conditions and the cohesive character of biofilms formed under more intense shaking conditions. In addition, this second optimization round was performed only with MRS broth, and the effect of medium replacement on *L. plantarum* biofilm growth was assessed. Comparing the results of the two optimizations, it is clear that replacing the culture medium every day enhanced the number of culturable cells and biomass amount, overcoming possible nutritional limitations, and the use of quasi-static conditions led to the formation of more robust biofilms.

Other research groups evaluated the biofilm-forming capacity of *L. plantarum* and showed that different strains of *L. plantarum* were able to grow as a biofilm in MRS broth on abiotic surfaces, such as polystyrene and glass [29,56,57,58,59]. Klimko et al. [60] investigated the ability of LAB to form biofilms on a hydrophobic carrier, and *L. plantarum* was revealed to be the most prolific biofilm-forming bacterium. Several factors, such as the temperature, availability of nutrients, pH, contact time with the surface, growth stage, and surface hydrophobicity, can affect biofilm development [61]. Fernández et al. [62] reported that the capacity of *L. plantarum* to form biofilms is highly affected by the composition of the culture medium, growth temperature, and time of incubation, as in our work. They found that *L. plantarum* biofilms typically contain proteins and/or proteinaceous material cementing the biofilm cells to the surface [62]. Several compounds are described to be directly related to the cell surface characteristics, such as lipoteichoic acid, outer membrane proteins and lipids, surface fibrils, various fimbriae, or polysaccharides [63,64]. Some authors suggested the existence of an S-layer, a mono-layer composed of identical proteins or glycoproteins, on the surface of specific *Lactobacillus* strains that are involved in the adhesion phenomenon [53]. Another key mechanism associated with biofilm formation is auto-aggregation. The well-known ability of probiotic strains to auto-aggregate might promote adhesion to host cells or polymeric surfaces [52,65,66]. The ability of microorganisms to adhere to certain substrata depends on van der Waals attraction forces, gravitational forces, steric interactions, protein adhesion, and electrostatic interactions, but one of the more important factors is the hydrophobicity of the cells [3,42,63]. The hydrophobicity of the cell surface depends on the surface components of the bacterial cells, such as proteins and polysaccharides, and the adhesion phenomenon is influenced by the contact between the cell wall and the surface of attachment [60,67]. Previous studies reported the hydrophilic character of *L. plantarum* [56,67], this being in accordance with the hydrophilicity level determined in the present work. Millsap et al. [56] also demonstrated that urine components can affect the ability of lactobacilli to adhere to substrata [56]. However, this was not verified in the present work, as the culturability of *L. plantarum* biofilms was constant over time when in contact with AUM, showing that this culture medium does not influence lactobacilli viability and stability. Besides the cell surface properties, the characteristics of the surface material play an important role in bacterial adhesion, including the chemical composition, physicochemical properties (surface charge, hydrophobicity, topography, and roughness), and physicomechanical parameters (elastic modulus and hardness) [9]. Thus, the auto-aggregation capacity and efficient production of extracellular polymeric substances and other cell components may be responsible for maintaining the *L. plantarum* population adhered to the silicone coupons [68,69,70,71].

Regarding the antibiofilm activity of *L. plantarum* biofilms, they significantly inhibited *E. coli* proliferation by reducing its culturability in 24-h biofilms. Some possible applications for this short-term catheterization period are procedures where indwelling urinary catheters are used to assess urine output and prevent postoperative urinary retention. For example, 24-h catheterization may be used routinely after common gynecological procedures [72]. Randomized controlled trials suggested that a removal time of the urinary catheter at ≤6 h postoperatively seems to be more beneficial than immediate or >6 h removal for patients undergoing gynecologic surgeries, decreasing both the ambulation time and hospital stay [73,74]. In fact, many clinical trials recommended the removal of urinary catheters within the first postoperative day (within a period ranging from 3 to 24 h) for many surgical interventions that do not require long catheterization periods, such as pelvic rectal or colorectal resection surgery, gynecological procedures (gynecological laparotomy, cesarean section, colposuspension, vaginal plastic surgery, vaginal prolapse surgery, and vaginal, laparoscopic or abdominal hysterectomy) or urological surgeries (transurethral resection of the prostate) [74,75,76,77,78,79,80,81,82,83]. For operative patients who have an indication for an indwelling catheter, the removal of the catheter should be done as soon as possible postoperatively, preferably within 24 h [84]. To the best of our knowledge, this is the first study evaluating the short-term activity of pre-formed *L. plantarum* biofilms against *E. coli* colonization. Previously, Jalilsood et al. [59] evaluated the ability of an *L. plantarum* isolate to form strong biofilms, and its inhibitory effect against numerous spoilage and pathogenic bacteria, but for longer periods. The *L. plantarum* biofilms were formed over 7 days and, when exposed to bacteria, they significantly reduced the number of pathogens for up to 6 days.

The competitive features of probiotics depend on the ability to produce and release antimicrobial substances, compete for adhesion sites on the surface, and compete for limited resources with the pathogens [57,85]. Thus, the antagonizing effect of *L. plantarum* may be associated with antimicrobial and/or anti-adhesive properties.

The antimicrobial activity is correlated essentially with the production of antimicrobial substances, such as organic acids, hydrogen peroxide, exopolysaccharides, biosurfactants or bacteriocins, that can hinder adhesion to surfaces and generate unfavorable environmental conditions [21,22]. The production of organic acids, such as lactic and acetic acids, is one of the major antibacterial mechanisms of lactobacilli [86]. Organic acids have been shown to effectively inhibit microbial growth and kill Gram-negative bacteria like *E. coli* by lowering the pH, causing cell membrane damage and the consequent leakage of intracellular material [52,87]. Lactobacilli may produce high concentrations of lactic acid and acetic acid, depending on their fermentative pathways and growth conditions. Teanpaisan et al. [88] reported that *Lactobacillus* strains increased their inhibitory activity when they were cultured in a medium with a higher glucose concentration, indicating that the availability of substrate for fermentation is one of the essential factors to exert inhibitory effects. MRS broth, the appropriate medium for *Lactobacillus* growth, contains a high glucose concentration, which enables both lactic and acetic acid production from glucose metabolism. However, in AUM, the concentration of glucose is residual, limiting the growth of *L. plantarum* and preventing medium acidification. Hence, other mechanisms of action besides organic acid production were probably implicated in the antimicrobial activity shown by *L. plantarum*.

The prevention of *E. coli* biofilm formation may be attributed to the bacteriocins produced by *L. plantarum*. Different studies demonstrated that the secretion of bacteriocins by lactobacilli can inhibit the growth of competitors [43,45,89,90,91]. Zalán et al. [92] demonstrated that *L. plantarum* seemed to be a good bacteriocin producer, and exhibited a remarkable inhibitory performance against several pathogens. Bacteriocins from *L. plantarum* have been shown to induce cell death through the inhibition of cell wall, nucleic acid and protein synthesis, or enzymatic activity [60]. Bacteriocins have several other proposed antimicrobial mechanisms, such as pore formation in the cell membrane, leading to cell leakage and consequent death [21,93].

Furthermore, the production of hydrogen peroxide by probiotics is very important because it has bactericidal effects on most pathogens [39]. *L. plantarum* was reported to have the ability to produce hydrogen peroxide [94], which can be toxic to organisms lacking hydrogen peroxide-scavenging enzymes [95]. The strong oxidizing effect of hydrogen peroxide on the bacterial cell, and the destruction of basic molecular structures of cell protein, is the basis of its bactericidal effect [96]. *E. coli* can protect itself from the oxidative stress of endogenous hydrogen peroxide through the action of peroxidases and catalases [97]. However, although *E. coli* tolerates low levels of intracellular hydrogen peroxide, if the concentration were allowed to rise much higher than 50 nM, vulnerable enzymes would quickly lose activity and the pathways to which they belong would cease to function [97]. Hydrogen peroxide can cause DNA damage and other uncharacterized damages at concentrations higher than 1 mM, and decrease the expression of virulence factors of *E. coli* [87,97,98,99]. Kang et al. [96] detected a concentration of 3.5 mM of hydrogen peroxide in the culture medium of a *Lactobacillus* strain and indicated that the main inhibitory factor against *E. coli* was hydrogen peroxide. Thus, although the antimicrobial mechanisms suggested here require further research, the synergistic action of secreted bacteriocins or bacteriocin-like proteins and hydrogen peroxide might be related to the bactericidal activity of *Lactobacillus* strains against uropathogens.

Regarding the anti-adhesive properties of *L. plantarum*, it is most likely to be of an interfacial nature, presumably by the modification of the surface energy of *E. coli* cells, preventing them from clumping and forming an organized network [100]. Previous studies have demonstrated that biosurfactants produced by LAB were able to inhibit pathogens’ adhesion to surfaces (including silicone-based materials) through both antimicrobial and anti-adhesive mechanisms [46,101,102,103,104], which can include the reduction of the hydrophobicity of surface substratum and consequently alter microbial adhesion, and the disruption of the cell membrane structure, leading to the release of cellular content and cell death [21,28,46,105]. In addition, the presence of exopolysaccharides may exert anti-adhesive effects by weakening cell surface modifications, interfering with cell-cell interactions and restricting the attachment of *E. coli* to the surface [106]. It was reported that exopolysaccharides produced by probiotics might have reduced QS signals involved in pathogenic biofilm formation [93]. Mahdhi et al. [107] proposed that exopolysaccharides produced by *L. plantarum* decreased cell surface hydrophobicity and indole production (a signal molecule involved in QS), hindering *E. coli* biofilm formation. In the present study, when *E. coli* was challenged with probiotic biofilms, it is believed that the inhibitory effect may also be associated with the integrity and cell culturability of *L. plantarum* biofilms, since the decreasing efficacy of *L. plantarum* biofilms at long periods (24 h) was accompanied by a decline in biovolume, biofilm thickness and number of *L. plantarum* culturable cells. This phenomenon may occur due to (1) the replacement of probiotic sessile cells by *E. coli*, (2) probiotic detachment from silicone surfaces, or (3) nutritional limitations resulting from the batch mode in which the assays were performed, which may lead to nutrient depletion (since *L. plantarum* has more restrictive growth requirements [31], and *E. coli* has a more efficient carbon and nitrogen metabolism under nutrient-limited conditions [108]). The differences in the free energy of adhesion also suggest that *E. coli* is less prone to adhere to *L. plantarum* biofilms than to silicone, which helps to explain the antibiofilm activity of *L. plantarum* and the preferential location of *E. coli* cells in the innermost layers of the biofilm.

The probiotic activity seems to be mainly related to bacterial interactions, the pre-colonization of adhesion sites by *L. plantarum* cells*,* and the production of substances that kill pathogens. The present work revealed that *L. plantarum* attachment on silicone-based surfaces may be a promising approach for preventing the formation of pathogenic biofilms.

## 4. Materials and Methods

### 4.1. Preparation of Silicone Surfaces

In order to mimic the typical surface material of urinary catheters, biofilm formation assays were performed on square silicone coupons with dimensions of 1 × 1 cm (Neves & Neves, Lda, Gondomar, Portugal) placed inside 12-well plates (VWR, Radnor, PA, USA). The coupons were firstly washed with 70% (*v*/*v*) ethanol (VWR, Radnor, PA, USA), dried at room temperature for 1 h, and then sterilized through ultraviolet (UV) radiation for 30 min [109]. Double-sided adhesive tape was placed in each plate well, sterilized with UV radiation for 30 min, and finally, the sterile coupons were glued in place.

### 4.2. Bacterial Strains and Culture Conditions

The probiotic strain used in this study was *Lactobacillus plantarum* (kindly provided by Dr. Mette Burmølle, University of Copenhagen, Copenhagen, Denmark). *L. plantarum* was evaluated in terms of its biofilm-forming capacity and antibiofilm and anti-adhesive activities against uropathogens in exclusion assays. This strain was preserved at −80 °C in MRS broth (Merck, Madrid, Spain) with 30% (*v*/*v*) glycerol, streaked on MRS agar (Sharlab, Barcelona, Spain) plates, and incubated for 48 h at 37 °C. The inoculum was prepared by collecting bacterial colonies from the MRS agar plate into 250 mL of MRS broth and incubating overnight at 37 °C in an orbital shaker at 120 rpm (Agitorb 200; Aralab, Rio de Mouro, Portugal). *Escherichia coli* CECT 434 expressing GFP was used as a model strain of uropathogenic bacteria to perform the antibiofilm assays. For the construction of *E. coli* CECT 434 GFP, the previously described pCM11 plasmid carrying the gene-encoding superfolder GFP (sGFP) and conferring ampicillin resistance was introduced in the CECT 434 strain by the heat shock method [110]. This bacterial strain was preserved at −80 °C in Luria-Bertani (LB) broth (Thermo Fisher Scientific, Waltham, MA, USA) containing 30% (*v*/*v*) glycerol, streaked on LB agar plates supplemented with ampicillin at 0.1 g·L^−1^ to maintain pCM11 in *E. coli* CECT 434, and incubated for 24 h at 37 °C. Single colonies were collected from LB agar plates, inoculated in 250 mL of AUM supplemented with ampicillin [111], and incubated overnight at 37 °C and 120 rpm to prepare a starting culture. AUM was used for *E. coli* growth since it provides nutritional conditions similar to those found in human urine.

### 4.3. Biofilm-Forming Capacity of L. plantarum

An initial screening of the biofilm-forming capacity of *L. plantarum* was performed, testing different culture media, hydrodynamic conditions, and periods of biofilm formation, in order to determine the optimal conditions to form more robust biofilms. These were further used in antibiofilm assays (Section 4.4). *L. plantarum,* grown overnight in MRS broth, was harvested by centrifugation at 3202× *g* for 10 min at 25 °C (Eppendorf Centrifuge 5810R; Eppendorf AG, Hamburg, Germany), and the final cell concentration was adjusted to an optical density at 610 nm (OD_610 nm_) of 0.7 in fresh AUM and MRS broth, equivalent to approximately 10^8^ colony-forming units per mL (CFU·mL^−1^), as confirmed by CFU counts. Biofilm formation assays were performed on silicone coupons placed inside sterile 12-well plates, where each well was loaded with 3 mL of *L. plantarum* suspension. The plates were incubated for 24, 48 and 72 h at 37 °C under static and high shaking (100 rpm) conditions. These conditions were tested because the colonization of urinary catheters by uropathogenic strains can occur in both hydrodynamic environments: static conditions mimic the extraluminal surfaces in the urethra [9], while high shaking conditions generate shear stresses similar to those found inside the urinary catheter due to the flow of urine [9].

A second screening experiment was performed, where *L. plantarum* biofilms were formed in MRS broth for 24, 48 and 72 h at 37 °C under quasi-static (40 rpm) and high shaking conditions, with replacement of the medium every day by the aspiration of supernatant from wells and the addition of fresh medium. The quasi-static conditions mimic the low shear stresses found on the outer side of urinary catheters and stents housed inside the bladder [9]. Negative controls with 3 mL of sterile AUM and MRS broth were prepared to evaluate the surface sterility throughout the experiments.

After incubation, the suspensions were removed from the wells, the biofilms were gently rinsed with sodium chloride solution (NaCl 8.5 g·L^−1^) to remove the non-adherent cells, and the biofilm amount and culturability were assessed by crystal violet (CV) staining (Section 4.6) and CFU counts (Section 4.5), respectively. All experiments included at least three independent biological replicates, and each replicate included two technical replicates.

### 4.4. Antibiofilm Assays

The antibiofilm assays followed an exclusion strategy [22] that consisted of the exposure of *L. plantarum* biofilms, pre-formed in the optimal cultivation conditions found in Section 2.1 (for 48 h in MRS broth under quasi-static conditions with medium replacement every 24 h), to *E. coli*. Briefly, *L. plantarum* biofilms were formed on silicone coupons placed inside 12-well plates, as described above. Afterward, cell suspensions were removed, and each well was inoculated with 3 mL of *E. coli* suspension. For this test, after overnight incubation, *E. coli* cells were harvested by centrifugation (3202× *g*, 10 min, 25 °C), the cell concentration was adjusted to an OD_610 nm_ of 0.15 in AUM, equivalent to a concentration of 10^8^ CFU·mL^−1^, and a 100-fold dilution was performed in order to obtain a final concentration of 10^6^ CFU·mL^−1^, a representative dose of the minimal bacterial numbers found in the urogenital area [9]. Two negative controls were prepared: the first by adding the *E. coli* suspension to bare silicone, and the second by adding sterile AUM to *L. plantarum* biofilms. Plates were incubated at 37 °C for periods of contact of 3, 6, 12 and 24 h under quasi-static conditions (40 rpm) and, at the end of each experimental period, biofilms were monitored as described below. The ability of *L. plantarum* biofilms to maintain their integrity in AUM and inhibit *E. coli* biofilm formation was also assessed by plate count enumeration and confocal laser scanning microscopy (CLSM). All experiments included at least three independent biological replicates, and each replicate included two technical replicates.

### 4.5. Colony-Forming Units (CFU) Enumeration

The number of biofilm culturable cells per cm^2^ of silicone was determined by CFU counts. After washing off the non-adhered cells, the coupons were transferred to 15 mL Falcon tubes with 2 mL of sodium chloride solution (NaCl 8.5 g·L^−1^). Biofilm cells were detached from the coupons by vortexing (ZX4; VELP Scientifica Srl, Usmate, Italy) for 2 min at full power, and the obtained biofilm cell suspensions were serially diluted in sodium chloride solution, plated on LB agar supplemented with ampicillin (for *E. coli*) and MRS agar (for *L. plantarum*), and incubated at 37 °C for 24 and 48 h, respectively, for CFU enumeration. The percentages of CFU reduction of *E. coli* and *L. plantarum* were estimated according to Equation (1):

(1) $$Reduction\;\left(\%\right)=\frac{CFU_{control}-CFU_{biofilm}}{CFU_{control}}\times 100$$

where $CFU_{control}$ corresponds to the culturable cells of *E. coli* on bare silicone or of *L. plantarum* biofilms exposed to sterile AUM, and $CFU_{biofilm}$ corresponds to culturable cells of *E. coli* or *L. plantarum* in biofilms developed on silicone coated with *L. plantarum*.

### 4.6. Crystal Violet (CV) Staining

The total biomass of *L. plantarum* biofilms was quantified using the CV staining method, which is based on the ability of the CV dye to color some components present in the biofilm matrix and be retained by the peptidoglycan wall of both living and dead bacterial cells [112]. The protocol was adapted from previous works [113,114]. Briefly, after washing the biofilms, the silicone coupons were transferred to 24-well plates (Thermo Fisher Scientific, Waltham, MA, USA) to quantify only the amount of biofilm formed on the coupon. Biofilms were fixed with 1 mL of 100% ethanol (VWR, Radnor, PA, USA), which was removed after 15 min of contact. Then, the wells were air-dried, and 1 mL of 1% (*v*/*v*) CV (Merck, Taufkirchen, Germany) aqueous solution was added to each well and incubated for 5 min. The dye bound to the biofilm was solubilized by adding 1 mL of 33% (*v*/*v*) acetic acid (VWR, Radnor, PA, USA) aqueous solution. Finally, 200 μL from each well was transferred to a 96-well polystyrene plate (VWR, Radnor, PA, USA), and the biofilm biomass was determined through its measured absorbance at 570 nm in a microtiter plate reader (SpectroStar Nano; BMG LABTECH, Ortenberg, Germany). When absorbance values exceeded 1, samples were diluted in 33% (*v*/*v*) acetic acid, and the resulting measurements were corrected for the dilution factor. Absorbance measurements were corrected by subtracting the average of the blank control (wells containing surfaces not exposed to bacteria). The biofilm amount was plotted as Abs _570 nm_ values.

### 4.7. Confocal Laser Scanning Microscopy (CLSM) Analysis

To assess the spatial organization of biofilms and extract their quantitative structural parameters, the two control groups (*E. coli* and *L. plantarum* single-species biofilms formed on bare silicone) and the treatment group (*E. coli* biofilms formed on silicone pre-coated with *L. plantarum* biofilms), corresponding to the experimental periods of 12 and 24 h, were visualized by CLSM. The *L. plantarum* on the biofilms was counterstained in red with 5 μM Syto61 (Invitrogen, Illkirch-Graffenstaden, France), a cell-permeant nucleic acid marker, for 10 min. Each stained coupon was inverted, mounted on a coverslip, and scanned using a 40× water objective (Leica HCX PL APO CS; Leica Microsystems GmbH, Wetzlar, Germany) in an inverted microscope Leica DMI6000-CS with a 488-nm argon laser and 633-nm helium-neon laser. The emitted fluorescence was recorded within the range of 500–580 nm to collect the GFP emission fluorescence and 640–730 nm to collect the Syto61 fluorescence. A minimum of five stacks of horizontal plane images (512 × 512 pixels, corresponding to 387.5 µm × 387.5 µm) with a z-step of 1 µm was acquired for each biofilm sample.

Three- and two-dimensional projections of the biofilm structures were reconstructed from the CLSM acquisitions, using the “Easy 3D” and “Section” functions of IMARIS 9.1 software (Bitplane AG, Zurich, Switzerland), respectively. The plug-in COMSTAT2 associated with the ImageJ software was used to determine the biovolume (µm^3^·µm^−2^), biofilm thickness (µm), and surface coverage (%) [115]. The Stack Profile tool provided by the LAS AF Lite software (Leica Microsystems GmbH) was also used to trace the intensity values of both fluorescence signals concerning the *z*-position.

### 4.8. Contact Angle Measurements, Hydrophobicity, and Free Energy of Adhesion between Bacteria and Substrates

The free energy of adhesion ($\Delta G_{adhesion}$) between *E. coli* and all tested substrates (bare silicone and silicone coated with *L. plantarum* biofilm) was assessed according to the procedure described by Alves et al. [49]. Lawns of *E. coli* were prepared to ascertain the bacterial surface hydrophobicity. Briefly, *E. coli* substrate was prepared by collecting bacterial cells from an overnight culture (grown under the conditions described in Section 4.4) on a cellulose membrane (pore diameter of 0.45 µm; Advantec, Japan) to a density of 1 × 10^8^ cell·mm^−2^. Strips (with a width of 1 cm) were cut from the membranes and fixed onto a glass slide for contact angle determination. *L. plantarum* biofilms were formed as described in Section 4.4.

The contact angles of the bacteria and the substrates were determined by the sessile drop method, using a contact angle meter (OCA 15 Plus; DataPhysics, Filderstadt; Germany). The surface tension components of the bacteria and the adhesion surfaces were obtained by measuring the contact angles with three pure liquids (*I*): water, formamide and α-bromonaphthalene (Sigma-Aldrich Co., Lisboa, Portugal). The surface tension components of the reference liquids were obtained from the literature [116]. Contact angle measurements were performed at room temperature (25 ± 2 °C) in three independent experiments, and at least 20 determinations for each liquid, material, and microorganism were made. Afterward, the hydrophobicity of the bacteria and the surfaces were evaluated using the method of van Oss et al. [117,118,119]. In this approach, the degree of hydrophobicity of a given material (*s*) (surface or bacteria) is expressed as the free energy of interaction between two entities of that material when immersed in water (*w*)—$\Delta G_{sws}$. If the interaction between the two entities is stronger than the interaction of each entity with water ($\Delta G_{sws}$ < 0 mJ·m^−2^), the material is considered hydrophobic. Conversely, if $\Delta G_{sws}$ > 0 mJ·m^−2^, the material is hydrophilic. $\Delta G_{sws}$ was calculated from the surface tension components of the interacting entities, according to Equation (2):

(2) $$\Delta G_{sws}=-2{\left(\sqrt{\gamma_{s}^{LW}}-\sqrt{\gamma_{w}^{LW}}\right)}^{2}+4\left(\sqrt{\gamma_{s}^{+}\;\gamma_{w}^{-}}+\sqrt{\gamma_{s}^{-}\;\gamma_{w}^{+}}-\sqrt{\gamma_{s}^{+}\;\gamma_{s}^{-}}-\sqrt{\gamma_{w}^{+}\;\gamma_{w}^{-}}\right)$$

where $\gamma^{LW}$, defined as $\gamma^{LW}=11.1\times {\left(1+\cos\theta_{B}\right)}^{2}$ where $\theta_{B}$ is the contact angle determined using α-bromonaphthalene, accounts for the Lifshitz–van der Waals component of the surface free energy, and $\gamma^{+}$ and $\gamma^{-}$ are the electron acceptor and electron donor parameters, respectively, of the Lewis acid–base component, $\gamma^{AB}$, with $\gamma^{AB}=2\sqrt{\;\gamma^{+}\;\gamma^{-}}$.

The surface tension components were estimated by the simultaneous resolution of three equations of the type of Equation (3):

(3) $$(1+\cos\theta_{I})\;\gamma_{s}^{TOT}=2\left(\sqrt{\gamma_{s}^{LW}\;\gamma_{I}^{LW}}+\sqrt{\gamma_{s}^{+}\;\gamma_{I}^{-}}+\sqrt{\gamma_{s}^{-}\;\gamma_{I}^{+}}\right)$$

where $\theta_{I}$ is the contact angle for each liquid, and $\gamma^{TOT}=\gamma^{LW}+\gamma^{AB}$.

When studying the interaction (free energy of adhesion) between *E. coli* (*b*) and the surfaces of adhesion (*s*) when immersed in water (*w*), the total interaction energy, $\Delta G_{adhesion}$, can be determined using Equation (4):

(4) $$\Delta G_{adhesion}=\gamma_{sb}^{LW}-\gamma_{sw}^{LW}-\gamma_{bw}^{LW}+2\left[\sqrt{\gamma_{w}^{+}}\;\left(\sqrt{\gamma_{s}^{-}}+\sqrt{\gamma_{b}^{-}}-\sqrt{\gamma_{w}^{-}}\right)+\sqrt{\gamma_{w}^{-}}\;\left(\sqrt{\gamma_{s}^{+}}+\sqrt{\gamma_{b}^{+}}-\sqrt{\gamma_{w}^{+}}\right)-\sqrt{\gamma_{s}^{+}\;\gamma_{b}^{-}}-\sqrt{\gamma_{s}^{-}\;\gamma_{b}^{+}}\right]$$

Thermodynamically, if $\Delta G_{adhesion}$ < 0 mJ·m^−2^, the adhesion of the bacteria to the substratum is favorable, whereas adhesion is not favorable if $\Delta G_{adhesion}\;$> 0 mJ·m^−2^.

### 4.9. Statistical Analysis

Statistical analysis was performed using the IBM SPSS Statistics version 26 for Windows (IBM SPSS, Inc., Chicago, IL, USA). Descriptive statistics were used to calculate the mean and SD (from at least three experiments with duplicates) for the number of culturable cells, biofilm mass, and quantitative CLSM analysis (biovolume and biofilm thickness). The homogeneity of variances and the normality of data were verified for all response variables tested, using the Kolmogorov–Smirnov and Shapiro–Wilk tests. Since the number of culturable cells, biofilm mass and CLSM parameters were not normally distributed, a nonparametric analysis using the Kruskal–Wallis test was performed to assess whether there were statistically significant differences among groups, and the Mann–Whitney test was conducted to determine the differences between those groups. Statistically significant differences were considered for *p*-values < 0.05, corresponding to a confidence level of 95%.

## 5. Conclusions

Overall, *L. plantarum* showed promising results against pathogenic biofilms developed on a polymeric surface. *L. plantarum* was able to form stable biofilms on silicone surfaces. Its biofilm-forming capacity was affected by the composition and frequency of culture medium replacement, hydrodynamic conditions, and the time of incubation. *L. plantarum* demonstrated good short-term activity in inhibiting *E. coli* adhesion to silicone. We suggested that the antibiofilm activity observed by *L. plantarum* biofilms was mainly attributable to the anti-adhesive properties associated with the competitive exclusion mechanism that prevents the adhesion of *E. coli* on the surfaces. The killing of *E. coli* may also have occurred via the production of antimicrobial compounds, such as bacteriocins, hydrogen peroxide, exopolysaccharides, and biosurfactants. Thus, coating the surface with probiotics may be a promising strategy to prevent the initial attachment of pathogens in silicone-based medical settings.

## Figures and Tables

**Table 1:** Contact angles with the three reference liquids (water, formamide, and α-bromonaphthalene), surface and bacteria hydrophobicity, and energy of *E. coli*–surface interaction.

Sample	Contact Angle (°)			Hydrophobicity (mJ·m^−2^)	*E. coli*–Surface Interaction (mJ·m^−2^)
	$\theta_{w}$	$\theta_{F}$	$\theta_{B}$	$∆G_{sws}$	$∆G_{adhesion}$
*Surface*					
Bare silicone	107.1 ± 4.9	100.7 ± 2.4	85.8 ± 3.7	−62.4	36.1
*L. plantarum* biofilm	34.9 ± 2.8	39.5 ± 4.9	23.1 ± 2.6	34.3	84.0
*Bacteria*					
*E. coli* CECT 434 GFP	19.4 ± 2.2	78.1 ± 5.5	62.2 ± 4.9	131.2	N.A.

Abbreviations: $\theta_{w}$—water contact angle; $\theta_{F}$—formamide contact angle; $\theta_{B}$—α-bromonaphthalene contact angle; $\Delta G_{sws}$—free energy of interaction between two entities of a given material (*s*) (surface or bacteria) when immersed in water (*w*); $\Delta G_{adhesion}$—free energy of adhesion between *E. coli* and the surfaces when immersed in water; N.A.—not applicable. Values are means ± SDs of three independent experiments.

## Data Availability

The data presented in this study are available on request from the corresponding author. The data are not publicly available yet as some data sets are being used for additional publications.
